# PD-1 inhibitors in endometrial cancer

**DOI:** 10.18632/oncotarget.22583

**Published:** 2017-11-21

**Authors:** Romualdo Barroso-Sousa, Patrick A. Ott

**Affiliations:** Patrick A. Ott: Department of Medical Oncology, Dana-Farber Cancer Institute, Boston, MA, USA; Harvard Medical School, Boston, MA, USA

**Keywords:** endometrial cancer, immunotherapy, PD-1 inhibition, microsatellite instability-high, POLE

Women with recurrent or metastatic endometrial carcinoma who experience disease progression following first-line therapy have limited treatment options and a poor prognosis. Therefore, new therapies are urgently needed for this disease. Recently, data published from a cohort of patients included in the phase Ib KEYNOTE-028 clinical trial (NCT02054806) that evaluated the activity of the PD-1 directed antibody pembrolizumab (10 mg/Kg every 2 weeks) in patients with PD-L1-positive advanced or metastatic endometrial cancer [1]. Among the 75 patients screened for PD-L1 expression, 36 (48.0%) had PD-L1-positive tumors, defined as membranous staining in at least 1% of the tumor and associated inflammatory cells or positive staining in the stroma. Twelve of the patients with PD-L1 positive tumors did not meet the eligibility criteria and were excluded; the remaining 24 patients were enrolled in the study. After a median follow-up of 10.9 months, confirmed partial responses were achieved in 3 patients (13.0%, 95% confidence interval = 2.8%-33.6%). Three additional patients (13.0%) had stable disease, with a median duration of 24.6 weeks. The safety profile was similar to other pembrolizumab studies, and overall, pembrolizumab was well tolerated in this patient population.

Although the sample size of this study is small, it is worth to highlight that: 1) all the responders had endometrioid histology (*n* = 17), with no objective responses among the other 6 patients with different subtypes; 2) there is promise for durable clinical activity for a subset of patients (26%) with heavily pretreated cancers; 62.5% of patients had received ≥ 2 previous lines of therapy for advanced disease; 3) the median duration of response was not reached, with 2 patients still experiencing sustained responses and receiving pembrolizumab at the time of the data cutoff; however, similar to other cancer subtypes in which PD-1 inhibitors have activity, these data suggest that most patients with advanced endometrial cancer do not derive benefit from PD-1 inhibition given as monotherapy, even those with PD-L1 positive tumors. Therefore, the search for predictive biomarkers of response that could help to select the best candidates for monotherapy is needed.

Genetic aberrations of protein coding DNA in tumor cells can generate mutated peptides potentially creating neoantigens that, after processing and presentation on HLA molecules can be recognized by cytotoxic T-cells [2]. High mutational burden has been linked to clinical efficacy of immune checkpoint inhibitors in melanoma, non-small cell lung cancer (NSCLC) , and other malignancies [2], and a higher mutational burden correlates with immune cytolytic activity across different human cancers [2]. Accordingly, molecular alterations that disrupt the capability of a proper DNA replication and are associated with an ultra-hyper-mutated phenotype have been emerging as predictive biomarkers of response to PD-1 inhibitors [3]. Such alterations include inactivating mutations in the exonuclease proofreading domains of POLE and POLD1 genes, triggering loss of the DNA mismatch repair proteins either due to inactivating mutations in mismatch repair (MMR) genes (MLH1, MSH2, MSH6, PMS2), or due to epigenetic alterations resulting in hypermethylation of the MLH1 promoter [3].

Data from The Cancer Genome Atlas demonstrated that endometrial cancer comprises a heterogeneous disease, with the identification of at least 4 molecular subtypes including the POLE ultramutated and the microsatellite instability (MSI) hyper mutated groups [4] .Consistent with the hypothesis that higher mutational/neoantigen burden is associated with increased immunogenicity of tumors, POLE ultra-mutated and MSI-high endometrial cancers are richly infiltrated with T-cell lymphocytes, have high PD-1 and PD-L1 expression, and, perhaps, are excellent candidates for PD-1/PD-L1 inhibitors [5]. Among the patients included in the KEYNOTE-028 trial, all but one of the 19 patients tested had MSI-low status. The one patient with MSI-high status had disease progression as best response. Genomic profiling of the tumor of a patient who experienced a partial response after treatment with pembrolizumab revealed the presence of a POLE mutation [5]. Other reports have shown that different tumors harboring POLE or POLD1 mutations are also associated with response to PD-1 inhibitors [6, 7]. The FDA recently approved the use of pembrolizumab for patients with MSI-high or deficient MMR tumors, independent of cancer type [3].

In our opinion the data from this relatively small cohort of patients with endometrial cancer from the KEYNOTE-028 study point at a signal of activity of pembrolizumab in this disease [1]. We do recommend that all patients with endometrial cancer undergo a validated test to determine their MSI status. POLE mutations as predictive biomarkers of response to PD-1inhibitors deserve further investigation.
